# Egg Production, Egg Development, and Mortality of Zoo‐Bred Ozark Hellbenders (*Cryptobranchus alleganiensis bishopi*)

**DOI:** 10.1002/zoo.21869

**Published:** 2024-11-26

**Authors:** D. Cristina Macklem, Lauren Augustine, Mark D. Wanner, Jeffery A. Ettling, Trisha Crabill, Amanda S. Pedigo, Chawna Schuette, Patty L. Ihrig‐Bueckendorf, Aja J. Martin, Katie R. Noble, Justin M. Elden, Jeffrey T. Briggler

**Affiliations:** ^1^ Saint Louis Zoo St. Louis Missouri USA; ^2^ Philadelphia Zoo Philadelphia Pennsylvania USA; ^3^ Smithsonian National Zoological Park Washington DC USA; ^4^ Brookfield Zoo Brookfield Illinois USA; ^5^ Jacksonville Zoo and Gardens Jacksonville Florida USA; ^6^ U.S. Fish and Wildlife Service Columbia Missouri USA; ^7^ No Leash Needed O'Fallon Illinois USA; ^8^ Missouri Department of Conservation Jefferson City Missouri USA

**Keywords:** conservation breeding, head‐starting, reproduction, salamander, species recovery plan

## Abstract

Populations of Ozark hellbenders (*Cryptobranchus alleganiensis bishopi*, Grobman 1943) in Missouri and Arkansas are federally listed as endangered. The Saint Louis Zoo WildCare Institute's Ron and Karen Goellner Center for Hellbender Conservation, in collaboration with the Missouri Department of Conservation and US Fish and Wildlife Service, has developed a sustainable conservation breeding and head‐starting program, a priority for species recovery. Using 9 years of program data, we examined various egg production, egg development, and mortality responses of Zoo‐bred Ozark hellbenders. Our results identified river of origin and breeding location as important predictors of egg production responses including average breeding female total lengths as well as brooding male clutch size, total egg count, and the estimated number of female clutches, respectively. We found that breeding group generation was a significant predictor of Zoo‐bred hellbender egg development responses with hellbenders from the first breeding group generation ovipositing later and producing eggs that hatch later and develop longer than hellbenders from the second‐generation breeding group. These responses are consistent with females from the first breeding group generation being larger at the time of reproduction. Breeding group generation was also a significant predictor of proportional egg and total mortality, while the proportion of hatchling mortality was best predicted by breeding location, and the proportion of larval mortality was best predicted by river of origin. Ultimately, our results provide baseline metrics for the program and identify areas for further inquiry to maximize the success of future conservation breeding and head‐starting efforts at the Zoo.

## Summary

We contributed to the continued success of the Saint Louis Zoo Ozark hellbender conservation breeding program by identifying biotic and abiotic characteristics related to egg production, egg development, and mortality of hellbenders at the Zoo.

## Graphical abstract

More mature first‐generation breeding Ozark hellbenders at the Saint Louis Zoo oviposit earlier and have eggs that develop longer and hatch later than the less mature second‐generation Ozark hellbenders, which are the offspring of the first‐generation and were reared at the Zoo to sexual maturity. Ozark hellbender embryos are also pictured.

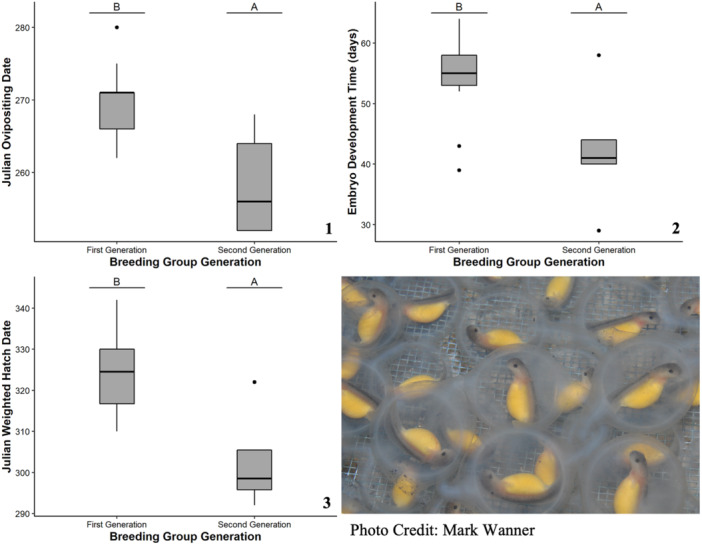

## 1 Introduction

Ozark hellbender (*Cryptobranchus alleganiensis bishopi*, Grobman 1943) populations have been threatened for decades and are declining throughout much of their historic range (Briggler et al. 2010; US Fish and Wildlife Service 2011, 2020). Primary threats likely include habitat disturbance, degradation, and loss, illegal collection, disease, reduced water quality, and predation by native and nonnative fish (Briggler et al. 2010; US Fish and Wildlife Service 2011, 2020). These drastic population declines have led to comprehensive conservation efforts by state and federal agencies as well as several partner organizations to halt and reverse current population trajectories.

Conservation breeding and head‐starting have been identified as a primary mechanism for augmenting wild Ozark hellbender populations in Missouri (Briggler et al. 2010, 2012; US Fish and Wildlife Service 2021). Conservation breeding and head‐starting programs have proven to be successful conservation tools for endangered amphibians (Browne et al. 2011; Harding, Griffiths, and Pavajeau 2016; Gratwicke and Murphy 2017; Silla and Byrne 2019). Most amphibians are highly fecund and can often be maintained under human care with relatively minimal space requirements and resources, unlike large or migratory mammals or birds (Browne et al. 2011; Harding, Griffiths, and Pavajeau 2016; Thomas et al. 2018). These initiatives also allow for controlled breeding, which, with appropriate protocols, can maximize genetic diversity for endangered species that are vulnerable to a loss of genetic diversity or inbreeding depression (Ralls, Ballou, and Templeton 1988; Crnokrak and Roff 1999; Browne et al. 2011; Gratwicke and Murphy 2017). Additionally, these conservation efforts provide a unique opportunity to learn more about the biology, physiology, life history, and behavior of species that could be data deficient, difficult to observe in the wild, or extinct in the wild (Browne et al. 2011; Harding, Griffiths, and Pavajeau 2016). Lastly, head‐starting initiatives can increase the success of individuals used for population augmentation by releasing them back into the wild at a size that is less vulnerable to potential causes of mortality (i.e., predation, infection, unfavorable environmental conditions, and stochastic events) or after reaching sexual maturity such that they can immediately contribute to recruitment in the wild population (Anderson, Hassinger, and Dalrymple 1971; Griffiths and Pavajeau 2008; Browne et al. 2011; Harding, Griffiths, and Pavajeau 2016; Thomas et al. 2018).

Due to the rapid decline of Ozark hellbenders in Missouri, a captive propagation and head‐starting program was initiated by the Saint Louis Zoo (Zoo) and Missouri Department of Conservation (MDC) in 2002 (Ettling and Briggler 2013). This initial partnership later developed into a much broader partnership among other state (Arkansas Game and Fish Commission) and federal (US Fish and Wildlife Service, US Forest Service, and National Park Service) agencies to develop an extensive head‐starting and conservation breeding program, complete with artificial streams, nest boxes, and integrated water quality and life support systems (Briggler et al. 2012; Ettling et al. 2013). The first Ozark hellbender larvae came to the Zoo in 2003, and the first 29 of these Zoo‐reared animals were released back into the wild starting in 2008 (Bodinof et al. 2012a, 2012b). In 2011, the Zoo became the first to successfully breed Ozark hellbenders (Ettling et al. 2013). Later that month, the Ozark hellbender subspecies was federally listed as endangered (US Fish and Wildlife Service 2011). In 2018, Ozark hellbenders that were originally bred at the Zoo successfully reproduced for the first time, creating a reproductively successful second‐generation breeding group. The Zoo's augmentation efforts continue to this day. By the end of 2020, 17 years into the program, the MDC had released 7975 Ozark hellbenders that were reared at the Zoo into four native Missouri rivers. The longevity and success of the propagation program at the Zoo provide a unique opportunity to learn more about the reproduction, development, and mortality of Zoo‐reared Ozark hellbenders, to examine the success of Zoo conservation breeding and head‐starting efforts for Ozark hellbenders, and to learn more about the biology of the subspecies.

Our objective was to evaluate the conservation breeding efforts of Ozark hellbenders at the Zoo. We accomplished this by investigating several factors that contribute to reproductive success, including egg production, egg development, and mortality. We hypothesized that biological factors related to the hellbender populations, the breeding individuals, and their reproductive output as well as external factors such as the Zoo habitat could influence the reproductive success factors we identified. Our goal was to define baseline metrics of success for the program, identify areas for improvement and/or further inquiry, and inform future strategies for the hellbender conservation breeding program at the Zoo.

## 2 Methods

### 2.1 Study Species and Husbandry

Ozark hellbenders are a fully aquatic salamander native to Missouri and Arkansas (U.S. Fish and Wildlife Service 2020). They inhabit cool, well‐oxygenated rivers and are usually found underneath large rock cover or within bedrock crevices (Briggler and Johnson 2021). In Missouri, the breeding season typically occurs between late September and late October (Peterson et al. 1983; Briggler and Johnson 2021). Reproduction in hellbenders usually includes males excavating nests under rock cover or within bedrock, female(s) ovipositing eggs within the nesting chambers, males fertilizing the eggs externally, and then males oxygenating the eggs and protecting them from predation throughout their development (Smith 1907; Nickerson and Mays 1973; Settle, Briggler, and Mathis 2018). Most hellbender eggs develop and hatch in approximately 45–75 days (Smith 1907; Green and Pauley 1987; Petranka 1998), and hellbenders typically reach sexual maturity at 5–8 years old (Bishop 1941; Dundee and Dundee 1965).

The Ron and Karen Goellner Center for Hellbender Conservation (hereafter: Center) in the Charles H. Hoessle Herpetarium at the Zoo houses and cares for Ozark hellbenders as a major component of the comprehensive recovery effort for the subspecies (Briggler et al. 2010, 2012). Given the Center's objective of population augmentation, the care provided by the Zoo is not intended to and cannot replicate a fully randomized experimental design, though husbandry protocols seek to provide consistent, standardized care. This is accomplished through the maintenance of three artificial streams designed to house adult hellbenders. One stream is located inside and measures 9.7 × 1.7 × 0.6 m deep, while two outdoor streams measure 11.3 × 1.5 × 1.4 m deep. These streams are designed to replicate natural conditions as closely as possible with recirculating flowing water, gravel bottoms, flat cover objects, artificial nest boxes, and tightly maintained water quality parameters (Ettling et al. 2013; Pedigo et al. 2021). The streams are also stocked with live food items, including crayfish, darters, sculpin, and/or minnows. See Pedigo et al. (2021) for more detailed hellbender husbandry protocols from the Zoo.

Each artificial stream at the Zoo houses a breeding group of Ozark hellbenders, which consists of two to four females and two to four males from the same river of origin to maintain the unique genetic lineages of the subspecies. Mitochondrial and genomic studies have shown low levels of within‐population variation and distinct genetic signatures between Ozark hellbender populations with hellbenders from the Current River (CR; Missouri) and Eleven Point River (EPR; Missouri and Arkansas) being genetically distinct from other populations of Ozark hellbenders such as those from the North Fork of the White River (NFWR; Missouri) (Sabatino and Routman 2009; Crowhurst et al. 2011; Tonione, Johnson, and Routman 2011; Hime 2017). Thus, while being classified as the same subspecies, the Zoo focused on maintaining these unique genetic lineages when establishing the first‐generation of Ozark hellbender breeding groups at the Zoo, which were composed of wild adults from the NFWR, EPR, and CR (Briggler et al. 2012).

Following oviposition and fertilization, eggs produced at the Zoo are collected from the nest boxes, hatched in incubation trays, and reared in closely monitored aquaria tanks (Briggler et al. 2012; Ettling et al. 2013; Pedigo et al. 2021). Without genetic testing, it is impossible to know with certainty the parentage of the eggs. However, within a reproductive year, the brooding male was always known. We assumed that eggs brooded by a male belonged to that male because wild Cryptobranchid males are typically found territorially guarding nests and tending to the developing eggs in the nest (Smith 1907; Settle, Briggler, and Mathis 2018; Unger et al. 2020, 2021). Moreover, other captive male Cryptobranchids that were known to have fertilized a particular clutch have shown nest fidelity and parental care behaviors to those clutches (Ettling et al. 2013; Luo et al. 2018). As a result, unless specified, references to clutch or clutch size refer to the eggs collected from and brooded by unique males in a given reproductive year.

### 2.2 Analysis

#### 2.2.1 Egg Production

To assess egg production of Zoo‐bred Ozark hellbenders, we examined the following response variables: the average total length (i.e., a measurement from the tip of the hellbender snout to the tip of the hellbender tail; hereafter: TL) of breeding group females, the number of clutches brooded by males, the size of clutches brooded by males, the total egg count for breeding groups, and the estimated number of and clutch sizes for clutches produced by females. We calculated the average TL of all females within a breeding group in a given reproductive year as a proxy for egg production capacity because it is unclear which, or how many, females contributed egg clutches in a given reproductive year. We quantified egg and clutch count variables based on the total number of eggs (jelly coat and ovum) oviposited by the breeding females that were ultimately removed by Zoo staff during a given breeding season. We note that for egg count responses, the number of eggs collected are likely underestimates of the true number of oviposited eggs because eggs are not removed from brooding males until a minimum of 12–14 days after oviposition as per the Zoo's husbandry protocols (Pedigo et al. 2021). During that time, it is possible for the brooding male, as well as other breeding group hellbenders, to cannibalize eggs, and for predation by fish and/or crayfish to occur in the artificial streams. However, due to standardized care and feeding, we assumed that the cannibalism and predation pressures were not biased for specific clutches or breeding groups. We quantified the number of eggs and clutches produced in several ways to better understand the contributions of males, females, and breeding groups as a whole. We calculated the number of brooding male clutches as the number of males brooding eggs from a given breeding group in a given reproductive year. We also calculated the brooding male clutch size as the number of eggs collected from each male in a given reproductive year. This includes eggs from all female contributors to that nest. We quantified the total egg production of each breeding group in a given year with the response variable, total egg count, which was a count of all collected eggs from a given breeding group in a reproductive year. Lastly, we quantified female contributions to egg production by estimating how many females were contributing egg clutches and how many eggs each female was contributing. In the wild, female Ozark hellbenders from Missouri were found to oviposit between 82 and 348 eggs (Briggler and Johnson 2021). Therefore, we calculated the number of female clutches based on the total egg count for a particular breeding group in a reproductive year and a 350‐egg maximum for an individual female. For example, any breeding group that produced greater than 350 eggs would be estimated as having two female clutches. We estimated female clutch size by taking the total egg count for a particular breeding group in a reproductive year and dividing that by the estimated number of female clutches. For example, if a breeding group produced 600 eggs, that would equal two female clutches with an estimated average of 300 eggs per clutch.

#### 2.2.2 Egg Development

Egg development response variables included ovipositing date, weighted hatch date, and egg development time. We calculated ovipositing date as the average day that eggs brooded by unique males were oviposited. Not all eggs hatch on the same day, and the peak of hatching does not necessarily occur on the mean hatch‐date for a clutch. Thus, we calculated a weighted hatch date, which weights the date by the number of eggs that hatch each day, to arrive at an overall hatch‐date for a given clutch. We note that zookeepers may manually hatch the eggs of struggling larvae to avoid death. We used the manual hatch date for these individuals in our weighted hatch date calculation and assumed that this practice was not performed in a biased manner. We calculated egg development time as the number of days from oviposition to the weighted hatch date for each clutch. In this context, egg development time is a proxy for the developmental stage at hatching such that shorter development times are indicative of a less advanced developmental stage at the time of hatching (Duellman and Trueb 1986).

#### 2.2.3 Mortality

We quantified the proportional mortality of Zoo‐bred Ozark hellbenders at three life stages: egg, hatchling, and larva. We used proportional mortality calculations to allow for comparison between clutches of different sizes. We calculated proportional mortality during the egg stage as any known egg death or egg removal weighted by the original clutch size. We quantified egg mortality as any egg deaths and/or removals due to infertility, infection, or congenital deformities that were incompatible with life from the time that eggs were collected from the nest boxes to the hatching date. We included infertile eggs within this count to account for the reproductive cost to the breeding adults as well as the reproductive failure associated with the investment. Furthermore, it is not possible to determine whether the eggs were fertilized and did not develop due to genetic incompatibilities or if they were truly infertile. We calculated proportional hatchling mortalities as any death post‐hatching up to 2 months (60 days) of age weighted by the original number of hatchlings. This life stage is particularly vulnerable to mortality because hatchlings are relatively sedentary, entirely reliant on their yolk sacs for nutrition, and continuing to experience morphological changes as they absorb the yolk sac (Smith 1907, 1912). We calculated proportional larval mortalities as any death occurring from 60 days old to 1 year post the weighted hatch date weighted by the original larval count. Lastly, we added all mortalities across all life stages weighted by the original clutch size for a proportional total mortality response variable.

#### 2.2.4 Analysis

We used an information theoretic approach to assess what influences egg production, egg development, and mortality of Zoo‐bred Ozark hellbenders (Burnham and Anderson 2002). We used data obtained from Zoo conservation breeding activities from 2012 to 2020 and chose to use a model selection process due to having a limited sample size for multivariate regressions. Thus, we created an a priori candidate model set of univariate models that aimed to clarify the importance of our predictive variables. We expected that river of origin, breeding group generation, breeding location, average female TLs, clutch size, and/or reproductive year could be important predictors of our response variables. We hypothesized that biological differences between hellbender river populations might contribute to differences in egg production, egg development, and mortality responses. We hypothesized that breeding group generation would influence our responses because the second‐generation breeding group at the Zoo was young, and the data we have represent the first reproductive events for these individuals, which we suspected could reduce egg production and development time while increasing mortality due to reduced resources to devote to reproduction (Duellman and Trueb 1986). We hypothesized that breeding location (i.e., indoor or outdoor artificial stream) could influence our responses due to differences in environmental conditions between the indoor and two outdoor artificial streams at the Zoo. We hypothesized that the average TL of breeding females could influence our responses because larger females have a greater capacity to produce eggs and provide resources to eggs (Salthe 1969; Topping and Ingersol 1981; Duellman and Trueb 1986; Browne et al. 2014). We hypothesized that clutch size could influence egg development and mortality responses due to differences in egg resource allocation as a result of tradeoffs between the number of eggs and the quality of yolk reserves (Duellman and Trueb 1986). Lastly, we hypothesized that reproductive year could influence our responses due to unintended differences in care, health, or environmental conditions between reproductive years. We ranked models using the Akaike Information Criterion corrected for finite sample sizes (AICc) (Akaike 1973, 1974; Hurvich and Tsai 1989). We identified top‐ranking models as having the lowest AICc score and being at least two ΔAICc smaller than the next top model (Burnham and Anderson 2002). Next, we assessed model assumptions for all top‐ranking models using Shapiro‐Wilk normality tests as well as Bartlett and Levene's tests for homogeneity of variance. For models that met assumptions, we performed type II ANOVA tests and Tukey post hoc tests to compare differences within predictor variables. For models that did not meet assumptions, we performed Kruskal‐Wallis rank sum tests and pairwise comparisons using Wilcoxon rank sum tests with a Bonferroni adjustment for post hoc testing. Data were analyzed using program R (version 3.6.3) through the RStudio (version 1.2.5033) interface.

## 3 Results

All data for the study were collected from 20 unique Zoo‐bred clutches that were laid between 2012 and 2020 (Table 1). Of the response variables where the clutch was the sample unit, including egg development responses, all proportional mortality responses, and brooding male clutch size, nine clutches were produced by EPR breeding groups, three were produced by CR breeding groups, and eight were produced by NFWR breeding groups. Of the 20 clutches, 15 were produced by first‐generation breeding groups and the remaining five were produced by second‐generation breeding groups. Sixteen clutches were laid in the two outdoor artificial streams, and four clutches were laid in the indoor artificial stream at the Zoo.

**Table 1:** Zoo‐bred Ozark hellbender clutch data organized by the river of origin of the breeding group adults with the reproductive year and individual clutch number listed. The breeding group generation^a^, breeding location^b^, average female total length^c^, ovipositing date^d^, weighted hatch date^e^, number of egg development days^f^, clutch size^g^, and total proportional mortality^h^ are listed for each clutch.

	Generation	Breeding location	Average female total length	Ovipositing date	Weighted hatch date	Development days	Clutch size	Total proportional mortality
Current River								
2012‐1	1	Outdoor	49.5	9/27/2012	11/22/2012	56	258	45%
2012‐2	1	Outdoor	49.5	9/27/2012	11/30/2012	64	401	63%
2012‐3	1	Outdoor	49.5	—	11/26/2012	—	409	72%
Eleven Point River								
2012‐1	1	Outdoor	43.5	9/18/2012	11/14/2012	57	211	71%
2012‐2	1	Outdoor	43.5	10/1/2012	12/1/2012	61	339	29%
2014‐1	1	Outdoor	41.3	9/23/2014	11/20/2014	58	293	35%
2015‐1	1	Outdoor	41.3	9/26/2015	11/19/2015	54	430	31%
2016‐1	1	Outdoor	43.0	9/19/2016	11/11/2016	53	235	15%
2018‐1	2	Outdoor	40.8	9/9/2018	10/19/2018	40	304	94%
2018‐2	2	Outdoor	40.8	9/9/2018	—	41	40	100%
2019‐1	2	Outdoor	42.4	9/21/2019	11/18/2019	58	647	75%
2020‐1	2	Outdoor	43.0	9/24/2020	10/23/2020	29	84	73%
North Fork River of the White River								
2012‐1	1	Indoor	51.0	9/27/2012	11/5/2012	39	560	—
2012‐2	1	Indoor	51.0	9/27/2012	11/9/2012	43	832	—
2013‐1	1	Indoor	50.7	9/28/2013	11/21/2013	54	414	47%
2013‐2	1	Indoor	50.7	9/29/2013	11/23/2013	55	1195	65%
2014‐1	1	Outdoor	50.8	9/19/2014	11/10/2014	52	771	43%
2015‐1	1	Outdoor	50.6	—	—	—	231	100%
2016‐1	1	Outdoor	48.7	10/6/2016	12/7/2016	62	165	78%
2020‐1	2	Outdoor	45.0	9/12/2020	10/26/2020	44	533	90%

^a^ Denotes whether a clutch was produced by the Saint Louis Zoo's first‐generation breeding group or second‐generation breeding group.

^b^ The location, either indoors or outdoors, of the artificial breeding streams at the Saint Louis Zoo.

^c^ The average total length of all females within a breeding group in a given reproductive year.

^d^ The average day that eggs brooded by unique males were oviposited.

^e^ A calculated hatch date for a clutch that weighs the number of eggs hatched each day during the hatching period.

^f^ The number of days from the oviposition date to the weighted hatch date for each clutch.

^g^ The number of eggs collected from a unique brooding male in a given reproductive year.

^h^ The proportion of all mortalities, up to 1‐year post the weighted hatch date, for a clutch weighted by the original clutch size.

The remaining egg production responses, including average female TL, number of brooding male clutches, total egg count, estimated number of female clutches, and estimated female clutch size, used breeding group reproduction events as the sample unit rather than clutch. Reproduction events occurred when unique breeding groups reproduced in a given year. Thus, these data represent seven unique EPR reproduction events, one unique CR reproduction event, and six unique NFWR reproduction events. First‐generation breeding group hellbenders were responsible for 10 reproduction events, while second‐generation breeding group hellbenders were responsible for the remaining four reproduction events. Twelve breeding events occurred in the two outdoor artificial streams, and two breeding events occurred in the indoor artificial stream.

### 3.1 Egg Production

**Figure 1:**
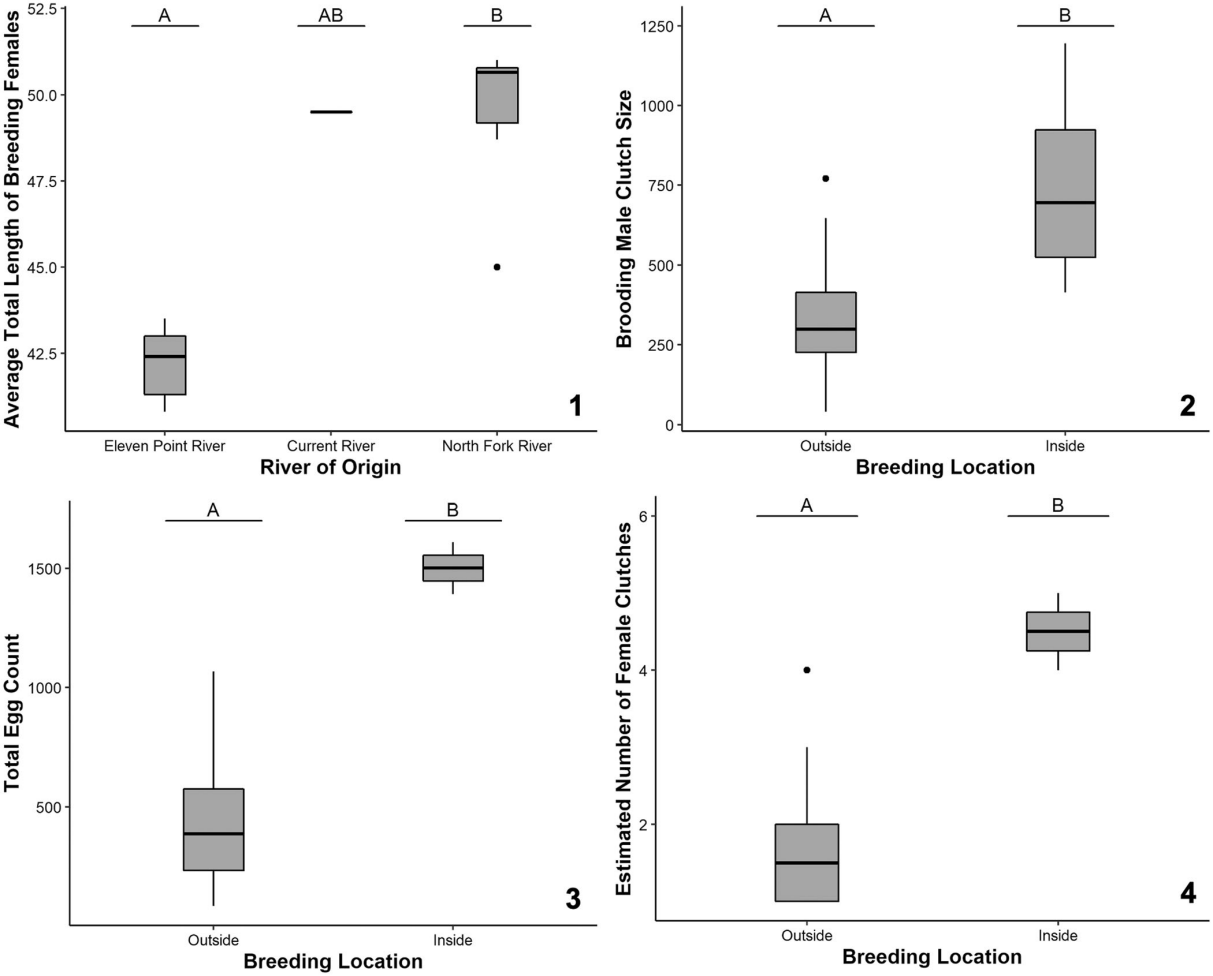
The Zoo‐bred Ozark hellbender egg production responses of average total length of breeding females (1), brooding male clutch size (2), total egg count (3), and estimated number of female clutches (4) as a function of significant predictive factors including river of origin and breeding location. Lettering over box plots denotes differences between factor levels with unique letters indicating significant differences, while multiple letters indicate values that are indistinguishable from either unique letter.

The average of average female TLs in each breeding group during a reproductive year was 45.8 cm with a range from 40.8 cm to 51 cm. The top‐ranking model for the average TL of each breeding group's females included the river of origin (Table 2). River of origin was significantly related to average female TLs (*χ*² = 9.91, *p* = 0.007). Female breeding group hellbenders from the NFWR had longer average TLs than female breeding group hellbenders from the EPR, and individuals from the CR had intermediate average TLs (Figure 1).

**Table 2:** Candidate egg production (average female total length, number of brooding male clutches, brooding male clutch size, total egg count, estimated number of female clutches, and estimated female clutch size), egg development (date of oviposition, weighted hatch date, and number of egg development days), and mortality (proportional egg mortality, proportional hatchling mortality, proportional larval mortality, proportional total mortality) model sets. For each model, the table reports the number of parameters (*k*), AIC value corrected for small samples size (AICc), as well as the calculated change in AICc (ΔAICc), and model weight (*w*).

	Intercept	River of origin	Generation	Breeding location	Average female total length	Clutch size	Reproductive year
*k*	1	3	2	2	2	2	8
*Egg production*							
Average female total length							
AICc	82.62	**62.58**	81.65	81.02	—	—	115.22
ΔAICc	20.04	**0.00**	19.07	18.44	—	—	52.64
*w*	0.00	**1.00**	0.00	0.00	—	—	0.00
Number of male brooded clutches							
AICc	30.80	**2741**	33.09	31.44	32.33	—	43.91
ΔAICc	3.39	**0.00**	5.68	4.03	4.92	—	16.50
*w*	0.13	**0.68**	0.04	0.09	0.06	—	0.00
Male brooded clutch size							
AICc	285.17	284.20	286.80	**278.26**	282.58	—	301.76
ΔAICc	6.91	5.94	8.54	**0.00**	4.32	—	23.51
*w*	0.03	0.04	0.01	**0.83**	0.09	—	0.00
Total egg count						—	
AICc	215.03	216.46	216.69	**201.97**	211.96	—	236.72
ΔAICc	13.06	14.49	14.73	**0.00**	9.99	—	34.76
*w*	0.00	0.00	0.00	**0.99**	0.01	—	0.00
Estimated number of female clutches							
AICc	51.44	52.76	52.38	**43.12**	47.38	—	74.27
ΔAICc	8.32	9.64	9.64	**0.00**	4.25	—	31.15
*w*	0.01	0.01	0.01	**0.86**	0.10	—	0.00
Estimated female clutch size							
AICc	162.80	168.76	165.54	**162.45**	165.48	—	191.37
	0.96*						
	0.27						
ΔAICc	0.35*	6.30	3.08	**0.00**	3.02	—	28.92
*w*	0.36	0.02	0.09	**0.43**	0.10	—	0.00
*Egg development*							
Date of ovipositing							
AICc	127.36	129.45	**118.98**	127.60	123.40	129.31	136.76
ΔAICc	8.38	10.47 00.01	**0.00**	8.61	4.42	10.33	17.78
*w*	0.01	0.00	**0.87**	0.01	0.10	0.00	0.00
Julian weighted hatch date							
AICc	148.23	150.64	**139.39**	150.77	148.87	150.78	158.04
ΔAICc	8.84	11.25	**0.00**	11.38	9.49	11.39	18.65
*w*	0.01	0.00	**0.97**	0.00	0.01	0.00	0.00
Development days							
AICc	135.535.	138.80	**130.63**	137.39	137.72	137.91	150.38
ΔAICc	4.90	8.17	**0.00**	6.76	7.09	7.28	19.75
*w*	0.07	0.01	**0.84**	0.03	0.02	0.02	0.00
*Mortality*							
Proportional egg mortality							
AICc	15.85	18.04	**10.32**	16.09	16.97	16.93	24.93
ΔAICc	5.53	7.72	**0.00**	5.76	6.65	6.60	14.60
*w*	0.05	0.02	**0.82**	0.05	0.03	0.03	0.00
Proportional hatchling mortality							
AICc	−16.40	−14.99	−16.04	**−21.49**	−13.88	−15.47	−15.41
ΔAICc	5.08	6.50	5.44	**0.00**	7.61	6.01	6.08
*w*	0.06	0.03	0.05	**0.77**	0.02	0.04	0.04
Proportional larval mortality							
AICc	−0.83	**−9.78**	−0.14	0.13	1.80	1.41	15.18
ΔAICc	8.95	**0.00**	9.65	9.91	11.58	11.19	94.96
*w*	0.01	**0.97**	0.01	0.01	0.00	0.00	0.00
Proportional total mortality							
AICc	5.49	10.01	**0.32**	7.88	8.02	7.73	24.46
ΔAICc	5.16	9.69	**0.00**	7.56	7.70	7.40	24.13
*w*	0.07	0.01	**0.87**	0.02	0.02	0.02	0.00

*Note:* Breeding Ozark hellbenders at the Saint Louis Zoo from the more mature first‐generation oviposit ealier and have eggs that models with the lowest ΔAICc and highest weights are bolded. Competing models are indicated by an asterisk (*).

The average number of brooding male clutches in a given reproductive year for a specific river of origin breeding group was 1.4 clutches, with a range from one to three clutches. The top‐ranking model for the number of brooding male clutches per breeding group per year included river of origin (Table 2). River of origin was not a significant predictor of the response variable (*χ*² = 3.66, *p* = 0.160).

Average brooding male clutch size during a reproductive year was 418 eggs, with a range from 40 to 1195 eggs. The top‐ranking model for brooding male clutch sizes included breeding location (Table 2). Breeding location was a significant predictor of brooding male clutch sizes (*F*_1,18_ = 10.79, *p* = 0.004), with male hellbenders from the indoor artificial stream brooding larger clutches than male hellbenders from the outdoor artificial streams (Figure 1).

The average total egg count for entire breeding groups in a reproductive year was 597 eggs, with a range from 84 to 1609 eggs. The top‐ranking model for total egg count for entire breeding groups in a reproductive year included breeding location (Table 2). Breeding location was a significant predictor of the response variable (*F*_1,12_ = 25.15, *p* < 0.001), with the hellbender breeding groups from the indoor artificial stream producing a higher total egg count per year relative to the hellbender breeding groups from the outdoor artificial streams (Figure 1).

We identified eight breeding group reproduction events with more than one presumed female contributor with three breeding groups producing more than 1000 eggs in a single reproductive year. The average estimated number of female clutches in a reproductive year was 2.1 clutches with a range from 1 to 5 clutches. The top‐ranking model for the estimated number of female‐laid clutches included breeding location (Table 2). Breeding location was a significant predictor of the response variable (*χ*² = 4.90, *p* = 0.027), with the expected number of female‐laid clutches being higher in the indoor artificial stream relative to the outdoor artificial streams (Figure 1).

The average estimated female clutch size was 259 eggs with a range from 84 to 348 eggs. The top‐ranking model for the estimated clutch size for female‐laid clutches included breeding location (Table 2). However, the intercept‐only model was within two ΔAICc (Table 2), which indicates that our predictive variables did not explain this response variable better than a null model. Breeding location was not a significant predictor of estimated female clutch size (*F*_1,12_ = 2.98, *p* = 0.110).

### 3.2 Egg Development

**Figure 2:**
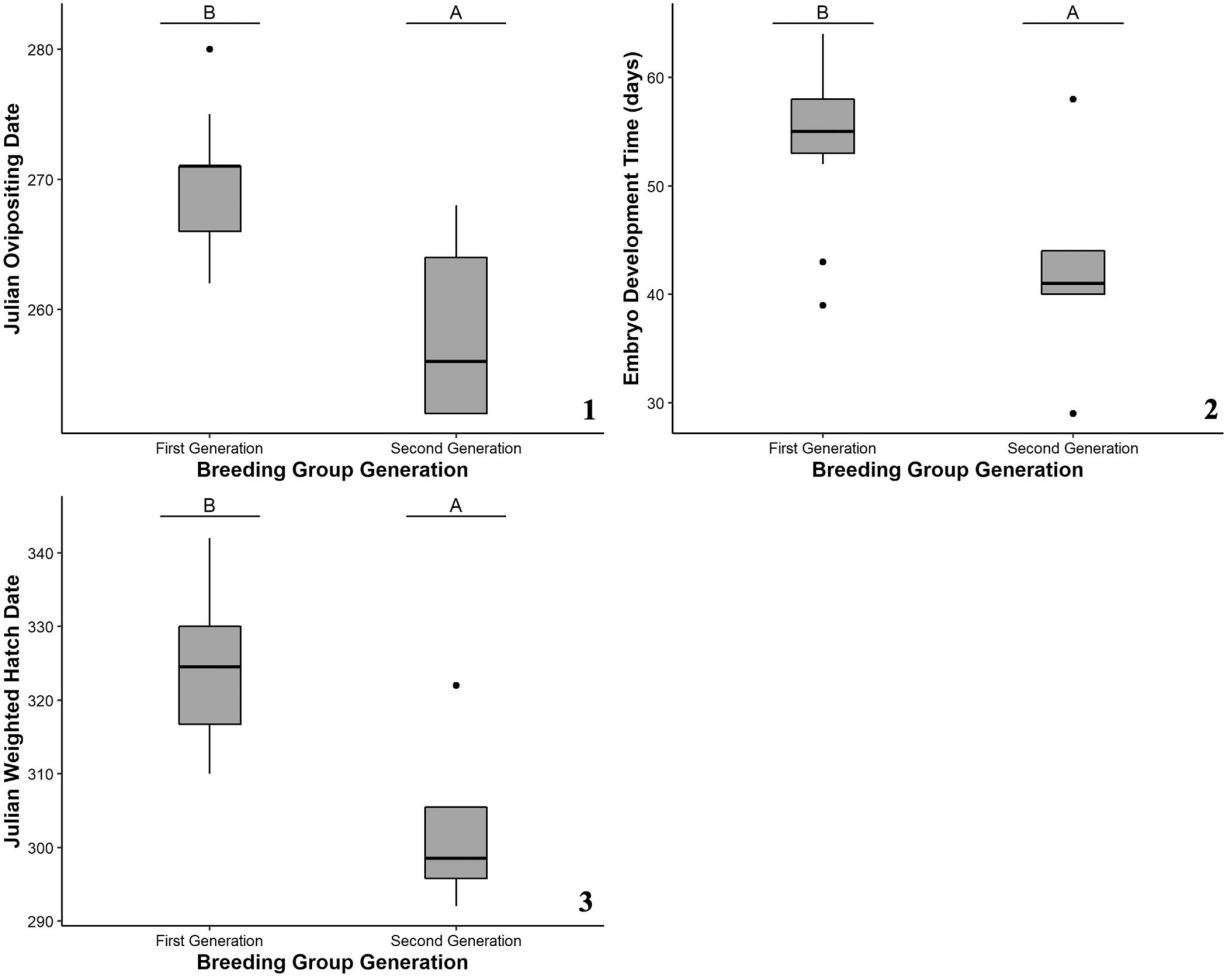
The Zoo‐bred Ozark hellbender egg development responses of Julian ovipositing date (1), Julian weighted hatch date (2), and embryo development time (3) as a function of the significant predictive factor, breeding group generation. Lettering over box plots denotes differences between factor levels with unique letters indicating significant differences.

Breeding females at the Zoo, on average, oviposited eggs on September 23. The earliest ovipositing date was 9 September, and the latest ovipositing date was October 7. The top‐ranking model for ovipositing date included breeding group generation (Table 2). Ovipositing date was significantly related to breeding group generation (*F*_1,16_ = 13.36, *p* = 0.002), with hellbenders from the first‐generation breeding group having later ovipositing dates than those from the second‐generation breeding group (Figure 2).

Hellbenders bred at the Zoo, on average, had a weighted hatch date of 16 November. The earliest weighted hatch date was 19 October and the latest was 8 December. On average, one standard deviation around the weighted hatch date was 4 days. The minimum was 1.7 days, and the maximum was 8.6 days. The top‐ranking model for weighted hatch date included breeding group generation (Table 2). Weighted hatch date was significantly related to breeding group generation (*F*_1,16_ = 14.13, *p* = 0.002), with hellbenders from the first‐generation breeding group having later weighted hatch dates than those from the second‐generation breeding group (Figure 2).

From the ovipositing date to the weighted hatch date, eggs at the Zoo develop in 51 days, on average. The shortest development time was 29 days and the longest was 64 days. The top‐ranking model for the number of egg development days included breeding group generation (Table 2). The number of egg development days was significantly related to breeding group generation (*F*_1,16_ = 8.21, *p* = 0.011), with hellbenders from the first‐generation breeding group having a longer egg development than those from the second‐generation breeding group (Figure 2).

### 3.3 Mortality

**Figure 3:**
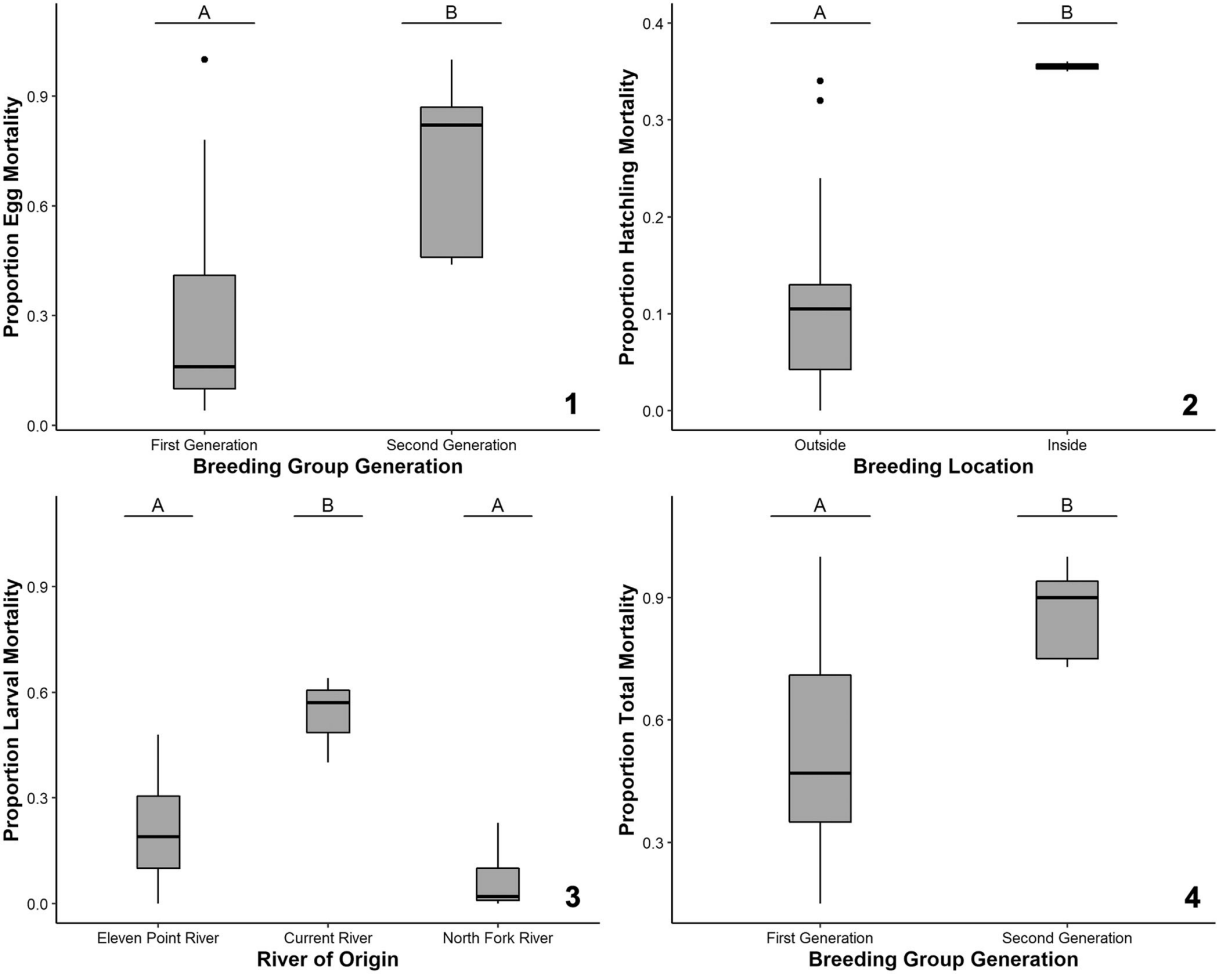
The mortality responses for Zoo‐bred Ozark hellbenders including proportional egg (1), hatchling (2), larval (3), and total (4) mortality as a function of the significant predictive factors of breeding group generation, breeding location, river origin, and breeding group generation, respectively. Lettering over box plots denotes differences between factor levels with unique letters indicating significant differences.

The average egg mortality of Ozark hellbenders bred at the Zoo was 40% of the original clutch size, with a minimum of 4% and a maximum of 100%. The top‐ranking model for the proportion of egg mortality included breeding group generation (Table 2), which was a significant predictor of the response variable (*χ*² = 5.98, *p* = 0.014). Hellbenders from the second‐generation breeding group had significantly higher proportional egg mortalities relative to hellbenders from the first‐generation breeding group (Figure 3).

The average hatchling mortality was 15% of the original hatchling number with a range from 0% to 36%. The top‐ranking model for the proportion of hatchling mortality included breeding location (Table 2). Breeding location was a significant predictor of the proportion of hatchling mortality (*χ*² = 4.97, *p* = 0.026). The proportion of hatchling mortality was higher for hellbenders bred in the indoor artificial stream compared to hellbenders bred in the two outdoor artificial streams (Figure 3).

The average larval mortality was 23% of the original number of larva with a range from 0% to 64%. The top‐ranking model for the proportion of larval mortality included river of origin (Table 2), which was a significant predictor of the response variable (*F*_2,13_ = 9.75, *p* = 0.003). Hellbenders from the EPR and NFWR had significantly lower proportions of larval mortality than CR individuals (Figure 3).

The average total mortality across life stages was 63% of the original clutch size with a range from 15% to 100%. The top‐ranking model for the proportion of total mortality included breeding group generation (Table 2), which was a significant predictor of the response variable (*F*_1,18_ = 8.56, *p* = 0.010). Hellbenders from the second‐generation breeding group had significantly higher proportional total mortalities relative to hellbenders from the first‐generation breeding group (Figure 3).

## 4 Discussion

Our study examined biological outcomes from 9 years of Ozark hellbender conservation breeding efforts by the Zoo and its partners. We successfully identified factors influencing the egg production, egg development, and mortality of Zoo‐bred Ozark hellbenders. We identified river of origin and breeding location as important predictors of some egg production responses, such as average breeding female TLs, brooding male clutch size, total breeding group egg counts, and the estimated number of female clutches. We also found that breeding group generation significantly influenced the egg development responses of ovipositing dates, weighted hatch date, and development days, with hellbenders from the first breeding group generation ovipositing and hatching later and developing for more time. Additionally, the most important predictors of mortality for Zoo‐bred Ozark hellbenders differed by life stage. Breeding group generation was a significant predictor of proportional egg mortality and proportional total mortality, while breeding location best predicted proportional hatchling mortality and river of origin best predicted proportional larval mortality. Ultimately, our results highlight areas for future exploration and monitoring opportunities of Zoo‐bred hellbenders to maximize the success of future conservation breeding and head‐starting efforts at the Zoo.

The results from our examination of egg production of Ozark hellbenders in the Zoo's breeding program contradicted our prediction. We anticipated that river of origin would be an important predictor for the average breeding female TLs because surveys of wild individuals and Zoo individuals indicate that NFWR individuals tend to be larger than CR or EPR individuals (Peterson et al. 1983, 1988; Wheeler et al. 2003; Macklem, et al. 2024). However, breeding location was the top‐ranking model for brooding male clutch size, total egg count, estimated number of female clutches, and estimated female clutch size. Moreover, the proportional hatchling mortality was higher for hellbenders from the indoor artificial stream than the outdoor artificial streams. These results could indicate that further research is needed to quantify and distinguish the features of the indoor and outdoor artificial streams that could influence egg production at the Zoo. For example, perhaps specific climactic or habitat configuration differences could support males brooding larger clutches, females ovipositing more eggs and clutches, and/or facilitate reproductive interactions between more males and females during the breeding season.

Alternatively, our finding that breeding location impacts egg production responses could be a statistical artifact of which breeding group occupied each artificial stream. Only breeding groups with individuals from the NFWR bred in the indoor artificial stream. While it is possible that the conditions of the indoor stream provided some unique increase in egg production responses to the NFWR hellbenders or that those hellbenders were exceptionally healthier or genetically more diverse than other breeding groups, we hypothesize that our results relate to the size and maturity of the NFWR breeding group that bred in the indoor artificial stream. Similar to studies from populations in the wild and at the Zoo (Peterson et al. 1983, 1988; Wheeler et al. 2003; Macklem, et al. 2024), the average TL of breeding group females from the NFWR was greater than that of the CR or EPR individuals at 49.7, 49.5, and 42.1 cm, respectively. This discrepancy increased when comparing the average TL of breeding group females from the NFWR that bred in the indoor artificial stream, 50.8 cm, relative to those that bred in an outdoor artificial stream, 49.0 cm. This difference in TLs was due to the transition from the first breeding group generation to the second breeding group generation after the breeding group was transferred to an outdoor artificial stream. Thus, the breeding location predictor variable is likely identifying differences in egg production as a result of its inclusion of the largest possible breeding group females kept at the Zoo. Larger and more mature females typically produce larger clutches (Salthe 1969; Topping and Ingersol 1981; Duellman and Trueb 1986; Nussbaum 1987; Browne et al. 2014), and the first‐generation NFWR breeding group produced more and larger clutches on average relative to the second‐generation NFWR breeding group, at 1.4 and 1 clutches and 595 and 533 eggs, respectively. This indicates that the breeding location predictor could primarily be identifying that larger, more mature NFWR breeding group individuals have larger brooding male clutches, total egg counts, and estimated numbers of female clutches relative to other breeding groups and breeding group generations, which is in accordance with scientific expectations about body size and egg production. Future research comparing the outcomes of other breeding groups in the indoor stream could help clarify the importance of breeding location.

While our egg production estimates of the number of female clutches and female clutch sizes are just approximations, they provide useful baseline data for the conservation breeding program. We found similar minimum, maximum, and average female egg counts to hellbenders in the wild using the 350‐egg maximum. We observed a minimum of 84 and a maximum of 348 eggs, similar to the 82 minimum and 348 egg maximum observed in wild Missouri populations (Briggler and Johnson 2021). Our estimated average female contribution of 259 eggs also falls within the two average estimates we have for female Ozark hellbenders in Missouri of 217 and 270 eggs (Nickerson and Mays 1973; Briggler and Johnson 2021). However, on three occasions, the estimated number of female clutches exceeded the number of breeding females in a particular breeding group. The number of eggs laid on these occasions suggests that the females were laying more than 350 eggs on average, which exceeds the known 348‐egg maximum observed in the wild. This could be feasible because egg predation and cannibalism may be minimized in the Zoo relative to the wild, which could lead to underestimations of wild clutches. Hellbenders at the Zoo also have easily accessible and consistently available food, which could eliminate periods of food scarcity, minimize foraging activity, and allow for greater allocation of energy and resources to reproduction relative to wild hellbenders. These differences might make them more capable of producing clutches near their maximum egg production potential (Salthe 1969; Duellman and Trueb 1986). Additionally, the average number of mature eggs in the ovaries of Ozark hellbenders has been approximated at 447 (Topping and Ingersol 1981), which indicates that Ozark hellbenders have the capacity to produce greater than 350 eggs in a given reproductive year. Contrarily, there were two instances where the oviposition pattern with males in the breeding group was suggestive of more female contributors than our estimated number of female clutches, which would indicate that each female was likely ovipositing substantially fewer than 350 eggs. This type of underestimation is expected when using a maximum egg production value, as females may not produce eggs at their maximum capacity every year. While the use of a 350‐egg maximum is imperfect, it can provide useful estimates on what percentage of females within a breeding group are likely contributing to egg production each year and a means to estimate how the average number of eggs produced by breeding females shifts through time, both of which are potentially important baselines to compare the success of different breeding groups and breeding group generations. Moreover, these estimations may be some of the only available approximations the Zoo has to assess these important reproductive parameters retroactively, given that parentage is uncertain. As more is learned about Ozark hellbender breeding at the Zoo, these estimates and the methods used to calculate these values can be adjusted.

Zoo breeding group generation strongly influenced all egg development responses and proportional egg and total mortality responses. Our original hypothesis that second‐generation individuals were likely to have eggs with shorter development times and higher proportional mortalities due to reduced female size and, thus, reduced accumulation of resources for successful reproduction is supported by our data and the literature. The biological relationship between female size and clutch size, ovum size, and egg development time is well documented, with larger females tending to produce larger clutches, larger ovums, and eggs with longer development times that hatch at more advanced developmental stages (Salthe 1969; Topping and Ingersol 1981; Duellman and Trueb 1986; Nussbaum 1987; Browne et al. 2014). First‐generation breeding females had average TLs of 47.0 cm compared to 42.8 cm for second‐generation breeding females. Moreover, hellbenders from the first‐generation breeding group produced more eggs each year, on average, than the second‐generation breeding group, with 674 and 402 eggs, respectively. Additionally, hellbenders from the first‐generation breeding group had longer egg development periods with delayed ovipositing and weighted hatch dates relative to hellbenders from the second‐generation breeding group. On average, eggs from the first‐generation breeding group hatched in 54.5 days, while eggs from the second‐generation breeding group hatched in 42.4 days. In addition to potential differences in female contributions, we also hypothesize that larger, older males might have contributed to the higher egg survival of hellbenders from the first‐generation breeding group due to increased resources to allocate toward reproduction and parental care. Sperm production and mating can be costly for polygynous amphibians (Gibbons and McCarthy 1986; Hettyey et al. 2009), but larger amphibian and fish males tend to produce more sperm (O'Dea, Jennions, and Head 2014), be more successful at mating (Le Jacques and Lodé 2003) and fertilizing eggs (Gibbons and McCarthy 1986), and have eggs with higher hatching success (Le Jacques and Lodé 2003). In our case, it is possible that the first‐generation males might have increased sperm numbers, be more successful at mating and fertilizing eggs, and/or be better able to brood the eggs for the entire duration of their extended development, defend the eggs from predation, and perform energetically costly behaviors such as egg fanning and agitation than younger, smaller males. While we could not quantify differences in parental care because male hellbenders at the Zoo don't provide parental care for the duration of egg development, we did find evidence to suggest that second‐generation males were less successful at fertilizing the eggs. In those cases, we observed clutches being brooded by males where all or most eggs did not appear to develop at all, which could be indicative of a failure to fertilize the eggs. These cases contributed to the higher proportional egg mortality we observed for the second‐generation breeding group. Whether due to having larger, older females, males, or both, we observed significant differences in egg success with higher proportional egg survival for hellbenders from the first‐generation breeding groups than the second‐generation breeding group.

Prolonged egg development, like we observed in hellbenders produced by the first breeding group generation, often has cascading benefits to the eggs, which starts with hatching at a larger size and at a more advanced developmental stage (Salthe 1969; Kaplan and Kaplan 1980; Duellman and Trueb 1986). This is particularly important in the wild during the hatchling stage when mortalities are often elevated due to high levels of predation (Pagnucco, Paszkowski, and Scrimgeour 2011; Drake et al. 2014; Davenport et al. 2017), increased competition for limited food and shelter resources (Wilbur 1976, 1977; Werner 1986; Vonesh and De la Cruz 2002; Soteropoulos et al. 2014; Rasmussen and Rudolf 2015), exposure to disease (Garner et al. 2009; Fernández‐Benéitez et al. 2011); and vulnerability to environmental conditions and anthropogenic threats (Petranka and Sih 1986; Rogers and Chalcraft 2008; Di Minin and Griffiths 2011; Lowe et al. 2019; Cayuela et al. 2015; Reinhardt et al. 2018). Thus, the benefits of prolonged egg development and hatching at an advanced developmental stage can include reduced threats of predation, increased availability of food resources for hatchlings, and accelerated larval growth, ultimately leading to an amplification of earlier differences in size with time (Kaplan and Kaplan 1980; Sih and Moore 1993; Moore, Newton, and Sih 1996; Pagnucco, Paszkowski, and Scrimgeour 2011). These long‐term benefits could explain our finding that total proportional mortality was higher for hellbenders from the second‐generation breeding group. Alternatively, this result could indicate a strong influence of egg success on overall clutch success in the first year of life. In the future, we could examine the impacts of prolonged development on the post‐release success of reintroduced hellbenders.

We note that temperature and other environmental conditions can also play a significant role in amphibian reproduction, development, and survival (Duellman and Trueb 1986). The temperatures of the indoor and two outdoor artificial streams are fluctuated to mimic their rivers of origin but other ambient conditions are not controlled for (Pedigo et al. 2021). Thus, ambient temperatures and other environmental variables were variable during the breeding season and the minimum 12–14 days that the eggs remain in the artificial streams before being moved to the egg incubation trays. Our inclusion of Reproductive Year as a predictor variable in our model set was intended to identify potential differences in environmental conditions during that time period that were substantial enough to influence the reproductive variables we measured. Given that Reproductive Year was not a top model for any of our response variables, it is unlikely that these differences in environmental conditions are the primary driver of the results we observed.

In addition to breeding group generation influencing proportional egg and total mortality and breeding location influencing proportional hatchling mortality, we found that proportional larval mortality was best explained by the river of origin. Specifically, we observed higher mortality in CR individuals relative to EPR or NFWR individuals. This was an unexpected result that may suggest a negative population‐specific response to the conditions at the Zoo or arbitrarily poor reproductive success for the specific individuals in the breeding group. For example, this result could indicate reduced genetic fitness of the CR breeding group. However, only one CR breeding group was included in our analysis, and it is possible, due to the generally low genetic diversity of Ozark hellbender populations (Sabatino and Routman 2009; Crowhurst et al. 2011; Tonione, Johnson, and Routman 2011; Hime 2017), that this breeding group had particularly reduced genetic fitness by chance.

The average total mortality across all life stages (i.e., egg, hatchling, and larvae) was 63% with a range of 15% to 100%. Although these results may seem high, they take into account the entirety of hellbender egg clutches produced at the Zoo from 2012 to 2020, including occurrences of total or partial infertility and individual instances of humane euthanasia due to congenital deformities. For example, our inclusion of infertile eggs considerably increases the total egg mortality counts. Additionally, the humane euthanasia of hatchlings and larva with complications (e.g., spinal issues, bloating, anorexia, etc.) that are not ideal for long‐term husbandry investments or augmentation into wild populations further increases mortality in these life stages. Mortality rates should also decline as the second‐generation breeding groups mature. Our data set included the first breeding attempts of the newly sexually mature individuals that were reared from eggs at the Zoo. We anticipate that as these individuals become more mature and experienced breeders, the mortality rates of their eggs, hatchlings, and larva will decrease and more closely resemble the mortality rates of hellbenders produced by the first‐generation breeding group. Taking all of these factors into account, the average total mortality rate of Zoo‐reared hellbenders to age one is substantially lower than the 90% estimated mortality rate for hellbenders under natural conditions in the wild (Unger, Sutton, and Williams 2013), which ultimately contributes to more mature hellbenders augmenting wild populations. Considerable information has been gained through trial and error over the years to propagate and augment wild populations of hellbenders in Missouri, and we continue to advance our knowledge to conserve this subspecies.

Our study analyzed data from a long‐term Ozark hellbender conservation breeding and head‐starting program at the Zoo. This research established baseline reproduction, egg development, and mortality data as well as identified areas for future inquiry and methods to monitor the long‐term success of the Ozark hellbender conservation breeding program. Such programs provide important opportunities for zoos and other institutions to identify, monitor, and examine novel biological, behavioral, or natural history data. In addition to improving *ex‐situ* care and husbandry practices, collecting and publishing this baseline data can contribute to in‐situ conservation and management efforts for this endangered species.

## Ethics Statement

Research was conducted in compliance with applicable animal care guidelines and appropriate permits. Hellbender eggs from the wild were collected by J. Briggler of the Missouri Department of Conservation under the authority of the Wildlife Code of Missouri and federal permit.

## Conflicts of Interest

The authors declare that there are no conflicts of interest.

## Data Availability

The data are not publicly available due to conservation concerns for the species.

## References

[zoo21869-bib-0001] Akaike, H. 1973. “Information Theory and an Extension of the Maximum Likelihood Principle.” In Proceedings of the Second International Symposium on Information Theory, edited by B. N. Petrov , and F. Csaki , 267–281. Budapest: Akademiai Kiado.

[zoo21869-bib-0002] Akaike, H. 1974. “A New Look at the Statistical Model Identification.” IEEE Transactions on Automatic Control 19: 716–723.

[zoo21869-bib-0003] Anderson, J. D. , D. D. Hassinger , and G. H. Dalrymple . 1971. “Natural Mortality of Eggs and Larvae of *Ambystoma t. tigrinum* .” Ecology 52, no. 6: 1107–1112.

[zoo21869-bib-0004] Bishop, S. C. 1941. “The Salamanders of New York.” New York State Museum Bulletin 324: 1–365.

[zoo21869-bib-0005] Bodinof, C. M. , J. T. Briggler , R. E. Junge , et al. 2012a. “Postrelease Movements of Captive‐Reared Ozark Hellbenders (*Cryptobranchus alleganiensis bishopi*) Following Translocation to the Wild.” Herpetologica 68, no. 2: 160–173.

[zoo21869-bib-0006] Bodinof, C. M. , J. T. Briggler , R. E. Junge , et al. 2012b. “Survival and Body Condition of Captive‐Reared Juvenile Ozark Hellbenders (*Cryptobranchus alleganiensis bishopi*) Following Translocation to the Wild.” Copeia 2012, no. 1: 150–159.

[zoo21869-bib-0007] Briggler, J. T. , T. Crabill , K. J. Irwin , et al. 2012. Propagation, Augmentation, and Reintroduction Plan for the Ozark hellbender (*Cryptobranchus alleganiensis bishopi*). Jefferson City, MO, USA: Ozark Hellbender Propagation Committee.

[zoo21869-bib-0008] Briggler, J. T. , T. Crabill , K. J. Irwin , C. Davidson , J. Utrup , and A. Salveter . 2010. Hellbender Conservation Strategy: An Action Plan for the Recovery of the Ozark and Eastern Hellbender in the Ozark Highlands of Missouri and Arkansas. Jefferson City, MO, USA: Ozark Hellbender Working Group.

[zoo21869-bib-0009] Briggler, J. T. , and T. R. Johnson . 2021. “Family Cryptobranchidae, Hellbenders and Giant Salamanders.” In The Amphibians and Reptiles of Missouri (revised and expanded), 3rd ed., edited by L. Archer , 48–50. Jefferson City, MO, USA: Missouri Department of Conservation.

[zoo21869-bib-0010] Browne, R. K. , H. Li , Z. Wang , et al. 2014. “The Giant Salamanders (Cryptobranchidae): Part B. Biogeography, Ecology and Reproduction.” Amphibian and Reptile Conservation 5, no. 4: 30–50.

[zoo21869-bib-0011] Browne, R. K. , K. Wolfram , G. García , M. F. Bagaturov , and Z. J. J. M. Pereboom . 2011. “Zoo‐Based Amphibian Research and Conservation Breeding Programs.” Amphibian and Reptile Conservation 5, no. 3: 1–14 (e28).

[zoo21869-bib-0012] Burnham, K. P. , and D. R. Anderson . 2002. Model Selection and Multimodel Inference: A Practical Information‐Theoretic Approach, 2nd ed. New York, USA: Springer.

[zoo21869-bib-0013] Cayuela, H. , D. Arsovski , S. Boitaud , et al. 2015. “Slow Life History and Rapid Extreme Flood: Demographic Mechanisms and Their Consequences on Population Viability in a Threatened Amphibian.” Freshwater Biology 60: 2349–2361.

[zoo21869-bib-0014] Crnokrak, P. , and D. A. Roff . 1999. “Inbreeding Depression in the Wild.” Heredity 83: 260–270. 10504423 10.1038/sj.hdy.6885530

[zoo21869-bib-0015] Crowhurst, R. S. , K. M. Faries , J. Collantes , J. T. Briggler , J. B. Koppelman , and L. S. Eggert . 2011. “Genetic Relationships of Hellbenders in the Ozark Highlands of Missouri and Conservation Implications for the Ozark Subspecies (*Cryptobranchus alleganiensis bishopi*).” Conservation Genetics 12, no. 3: 637–646. 10.1007/s10592-010-0170-0.

[zoo21869-bib-0016] Davenport, J. M. , M. E. Hampson , A. B. King , and S. C. Bishir . 2017. “The Effects of Sunfish on Spotted Salamander Oviposition, Hatching Time, and Larval Survival.” Amphibia‐Reptilia 38: 327–337.

[zoo21869-bib-0017] Di Minin, E. , and R. A. Griffiths . 2011. “Viability Analysis of a Threatened Amphibian Population: Modelling the Past, Present and Future.” Ecography 34: 162–169.

[zoo21869-bib-0018] Duellman, W. E. , and L. Trueb . 1986. Biology of Amphibians. New York, USA: McGraw‐Hill.

[zoo21869-bib-0019] Dundee, H. A. , and D. S. Dundee . 1965. “Observations on the Systematics and Ecology of *Cryptobranchus* From the Ozark Plateaus of Missouri and Arkansas.” Copeia 1965, no. 3: 369–370. 10.2307/1440805.

[zoo21869-bib-0020] Drake, D. L. , T. L. Anderson , L. M. Smith , K. M. Lohraff , and R. D. Semlitsch . 2014. “Predation of Eggs and Recently Hatched Larvae of Endemic Ringed Salamanders (*Ambystoma annulatum*) by Native and Introduced Aquatic Predators.” Herpetologica 70, no. 4: 378–387.

[zoo21869-bib-0021] Ettling, J. A. , and J. T. Briggler . 2013. “Conservation Efforts for the Endangered Ozark Hellbender.” World Association of Zoos and Aquariums 14: 30–32.

[zoo21869-bib-0022] Ettling, J. , M. D. Wanner , C. D. Schuette , S. L. Armstrong , A. S. Pedigo , and J. T. Briggler . 2013. “Captive Reproduction and Husbandry of Adult Ozark Hellbenders, *Cryptobranchus alleganiensis bishopi* .” Herpetological Review 44, no. 4: 605–610.

[zoo21869-bib-0023] Fernández‐Benéitez, M. J. , M. E. Ortiz‐Santaliestra , M. Lizana , and J. Diéguez‐Uribeondo . 2011. “Differences in Susceptibility to Saprolegnia Infections Among Embryonic Stages of Two Anuran Species.” Oecologia 165, no. 3: 819–826. 21197546 10.1007/s00442-010-1889-5

[zoo21869-bib-0024] Garner, T. W. J. , S. Walker , J. Bosch , et al. 2009. “Life History Tradeoffs Influence Mortality Associated With the Amphibian Pathogen *Batrachochytrium dendrobatidis* .” Oikos 118, no. 5: 783–791.

[zoo21869-bib-0025] Gibbons, M. M. , and T. K. McCarthy . 1986. “The Reproductive Output of Frogs *Rana temporaria* (L.) With Particular Reference to Body Size and Age.” Journal of Zoology 209, no. 4: 579–593.

[zoo21869-bib-0026] Gratwicke, B. , and J. B. Murphy . 2017. “History of Captive Management and Conservation Amphibian Programs Mostly in Zoos and Aquariums. Part II‐Salamanders and Caecilians.” Herpetological Review 48, no. 2: 474–486.

[zoo21869-bib-0027] Griffiths, R. A. , and L. Pavajeau . 2008. “Captive Breeding, Reintroduction, and the Conservation of Amphibians.” Conservation Biology 22, no. 4: 852–861. 18616746 10.1111/j.1523-1739.2008.00967.x

[zoo21869-bib-0028] Green, N. B. , and T. K. Pauley . 1987. Amphibians and Reptiles in West Virginia. Pittsburgh, PA, USA: University of Pittsburgh Press.

[zoo21869-bib-0029] Harding, G. , R. A. Griffiths , and L. Pavajeau . 2016. “Developments in Amphibian Captive Breeding and Reintroduction Programs.” Conservation Biology 30, no. 2: 340–349. 10.1111/cobi.12612. 26306460

[zoo21869-bib-0030] Hettyey, A. , B. Vági , G. Hévizi , and J. Török . 2009. “Changes in Sperm Stores, Ejaculate Size, Fertilization Success, and Sexual Motivation over Repeated Matings in the Common Toad, *Bufo bufo* (Anura: Bufonidae): Reproductive Potential of Male *B. bufo* .” Biological Journal of the Linnean Society 96, no. 2: 361–371.

[zoo21869-bib-0031] Hime, P. M. 2017. Genomic Perspectives on Amphibian Evolution Across Multiple Phylogenetic Scales. University of Kentucky. 10.13023/ETD.2017.284.

[zoo21869-bib-0032] Hurvich, C. M. , and C.‐L. Tsai . 1989. “Regression and Time Series Model Selection in Small Samples.” Biometrika 76: 297–307.

[zoo21869-bib-0033] Kaplan, R. H. , and R. H. Kaplan . 1980. “The Implications of Ovum Size Variability for Offspring Fitness and Clutch Size Within Several Populations of Salamanders (*Ambystoma*).” Evolution 34: 51–64. 28563211 10.1111/j.1558-5646.1980.tb04788.x

[zoo21869-bib-0034] Le Jacques, D. , and T. Lodé . 2003. “Influence of Advertisement Calls on Reproductive Success in the Male Midwife Toad *Alytes obstetricans* .” Behaviour 140, no. 7: 885–898.

[zoo21869-bib-0035] Lowe, W. H. , L. K. Swartz , B. R. Addis , and G. E. Likens . 2019. “Hydrologic Variability Contributes to Reduced Survival Through Metamorphosis in a Stream Salamander.” Proceedings of the National Academy of Sciences of the United States of America 116, no. 39: 19563–19570. 31488710 10.1073/pnas.1908057116 PMC6765273

[zoo21869-bib-0036] Luo, Q. , F. Tong , Y. Song , H. Wang , M. Du , and H. Ji . 2018. “Observation of the Breeding Behavior of the Chinese Giant Salamander (*Andrias davidianus*) Using a Digital Monitoring System.” Animals 8, no. 161: 161. 30257506 10.3390/ani8100161 PMC6211081

[zoo21869-bib-0080] Macklem, D. C. , L. Augustine , M. D. Wanner , et al. 2024. “Growth and Mortality of Zoo‐Reared Ozark Hellbenders, *Cryptobranchus alleganiensis bishopi* (Grobman 1943).” *Zoo Biology*. 10.1002/zoo.21870. PMC11802483 39588554

[zoo21869-bib-0037] Moore, R. D. , B. Newton , and A. Sih . 1996. “Delayed Hatching as a Response of Streamside Salamander Eggs to Chemical Cues From Predatory Sunfish.” Oikos 77: 331–335.

[zoo21869-bib-0038] Nickerson, M. A. , and C. E. Mays . 1973. “The Hellbenders: North American “Giant Salamanders.” Milwaukee Public Museum Publications in Biology and Geology 1: 1–106.

[zoo21869-bib-0039] Nussbaum, R. A. 1987. “Parental Care and Egg Size in Salamanders: An Examination of the Safe Harbor Hypothesis.” Population Ecology 29, no. 1: 27–44.

[zoo21869-bib-0040] O'Dea, R. E. , M. D. Jennions , and M. L. Head . 2014. “Male Body Size and Condition Affects Sperm Number and Production Rates in Mosquitofish, *Gambusia holbrooki* .” Journal of Evolutionary Biology 27, no. 12: 2739–2744. 25403851 10.1111/jeb.12534

[zoo21869-bib-0041] Pagnucco, K. S. , C. A. Paszkowski , and G. J. Scrimgeour . 2011. “Wolf in Sheep's Clothing: Effects of Predation by Small‐Bodied Fish on Survival and Behaviour of Salamander Larvae.” Écoscience 18, no. 1: 70–78.

[zoo21869-bib-0042] Pedigo, A. S. , K. R. Noble , P. L. Ihrig‐Bueckendorf , et al. 2021. Saint Louis Zoo Hellbender Husbandry Manual. Saint Louis, MO, USA: Saint Louis Zoo WildCare Institute Ron Goellner Center for Hellbender Conservation.

[zoo21869-bib-0043] Peterson, C. L. , D. E. Metter , B. T. Miller , R. F. Wilkinson , and M. S. Topping . 1988. “Demography of the Hellbender *Cryptobranchus alleganiensis* in the Ozarks.” American Midland Naturalist 119, no. 2: 291–303.

[zoo21869-bib-0044] Peterson, C. L. , R. F. Wilkinson, Jr. , M. S. Topping , and D. E. Metter . 1983. “Age and Growth of the Ozark Hellbender (*Cryptobranchus alleganiensis bishopi*).” Copeia 1983, no. 1: 225–231.

[zoo21869-bib-0045] Petranka, J. W. 1998. “Family Cryptobranchidae: Hellbender and Giant Salamanders.” In Salamanders of the United States and Canada, edited by P. Strupp , 139–144. Washington, DC, USA: Smithsonian Institution Press.

[zoo21869-bib-0046] Petranka, J. W. , and A. Sih . 1986. “Environmental Instability, Competition, and Density‐Dependent Growth and Survivorship of a Stream‐Dwelling Salamander.” Ecology 67, no. 3: 729–736.

[zoo21869-bib-0047] Ralls, K. , J. D. Ballou , and A. Templeton . 1988. “Estimates of Lethal Equivalents and the Cost of Inbreeding in Mammals.” Conservation Biology 2, no. 2: 185–193.

[zoo21869-bib-0048] Rasmussen, N. L. , and V. H. W. Rudolf . 2015. “Phenological Synchronization Drives Demographic Rates of Populations.” Ecology 96, no. 7: 1754–1760. 26378297 10.1890/14-1919.1

[zoo21869-bib-0049] Reinhardt, T. , L. Baldauf , M. Ilić , and P. Fink . 2018. “Cast Away: Drift as the Main Determinant for Larval Survival in Western Fire Salamanders (*Salamandra salamandra*) in Headwater Streams.” Journal of Zoology 306: 171–179.

[zoo21869-bib-0050] Rogers, T. N. , and D. R. Chalcraft . 2008. “Pond Hydroperiod Alters the Effect of Density‐Dependent Processes on Larval Anurans.” Canadian Journal of Fisheries and Aquatic Sciences 65: 2761–2768.

[zoo21869-bib-0051] Sabatino, S. J. , and E. J. Routman . 2009. “Phylogeography and Conservation Genetics of the Hellbender Salamander (*Cryptobranchus alleganiensis*).” Conservation Genetics 10, no. 5: 1235–1246. 10.1007/s10592-008-9655-5.

[zoo21869-bib-0052] Salthe, S. N. 1969. “Reproductive Modes and the Number and Sizes of Ova in the Urodeles.” American Midland Naturalist 81: 467–490.

[zoo21869-bib-0053] Settle, R. A. , J. T. Briggler , and A. Mathis . 2018. “A Quantitative Field Study of Paternal Care in Ozark Hellbenders, North America's Giant Salamanders.” Journal of Ethology 36, no. 3: 235–242. 10.1007/s10164-018-0553-0.

[zoo21869-bib-0054] Sih, A. , and R. D. Moore . 1993. “Delayed Hatching of Salamander Eggs in Response to Enhanced Larval Predation Risk.” The American Naturalist 142: 947–960. 10.1086/285583 19425943

[zoo21869-bib-0055] Silla, A. J. , and P. G. Byrne . 2019. “The Role of Reproductive Technologies in Amphibian Conservation Breeding Programs.” Annual Review of Animal Biosciences 7: 499–519. 30359086 10.1146/annurev-animal-020518-115056

[zoo21869-bib-0056] Smith, B. G. 1907. “The Life History and Habits of *Cryptobranchus allegheniensis* .” The Biological Bulletin 13, no. 1: 5–39.

[zoo21869-bib-0057] Smith, B. G. 1912. “The Embryology of *Cryptobranchus allegheniensis*, Including Comparisons With Some Other Vertebrates I. Introduction; the History of the Egg before Cleavage.” Journal of Morphology 23, no. 1: 61–157.

[zoo21869-bib-0058] Soteropoulos, D. L. , S. L. Lance , R. W. Flynn , and D. E. Scott . 2014. “Effects of Copper Exposure on Hatching Success and Early Larval Survival in Marbled Salamanders, *Ambystoma opacum* .” Environmental Toxicology and Chemistry 33, no. 7: 1631–1637. 24729474 10.1002/etc.2601

[zoo21869-bib-0059] Thomas, P. , D. M. Boyer , D. A. Oehler , S. Silver , and L. Perrotti . 2018. “Headstarting as a Conservation Strategy for Threatened and Endangered Species.” In Scientific Foundations of Zoos and Aquariums: Their Role in Conservation and Research, edited by A. B. Kaufman , M. J. Bashaw , and T. L. Maple , 91–111. Cambridge, UK: Cambridge University Press.

[zoo21869-bib-0060] Tonione, M. , J. R. Johnson , and E. J. Routman . 2011. “Microsatellite Analysis Supports Mitochondrial Phylogeography of the Hellbender (*Cryptobranchus alleganiensis*).” Genetica 139, no. 2: 209–219. 10.1007/s10709-010-9538-9. 21161568

[zoo21869-bib-0061] Topping, M. S. , and C. A. Ingersol . 1981. “Fecundity in the Hellbender, *Cryptobranchus alleganiensis* .” Copeia 1981, no. 4: 873–876.

[zoo21869-bib-0062] Unger, S. , C. M. Bodinof Jachowski , L. Diaz , and L. A. Williams . 2020. “Shelter Guarding Behavior of the Eastern Hellbender (*Cryptobranchus alleganiensis alleganiensis*) in North Carolina Streams.” Southeastern Naturalist 19, no. 4: 742–758.

[zoo21869-bib-0063] Unger, S. , Z. C. Hull , L. Diaz , J. D. Groves , L. A. Williams , and C. M. Bodinof Jachowski . 2021. “Underwater Video Cameras Allow for Detection of North American Giant Salamanders (*Cryptobranchus alleganiensis alleganiensis*) in Both Captive and Wild Streams.” Aquaculture and Fisheries 6, no. 1: 106–110.

[zoo21869-bib-0064] Unger, S. D. , T. M. Sutton , and R. N. Williams . 2013. “Projected Population Persistence of Eastern Hellbenders (*Cryptobranchus alleganiensis alleganiensis*) Using a Stage‐Structured Life‐History Model and Population Viability Analysis.” Journal for Nature Conservation 21, no. 2013: 423–432.

[zoo21869-bib-0065] U.S. Fish and Wildlife Service . 2011. “Endangered and Threatened Wildlife and Plants; Endangered Status for the Ozark Hellbender Salamander.” Federal Register 76, no. 194: 61956–61978.

[zoo21869-bib-0066] US Fish and Wildlife Service . 2020. Biological Report for the Ozark Hellbender. Columbia, MO, USA: US Fish and Wildlife Service.

[zoo21869-bib-0067] US Fish and Wildlife Service . 2021. Recovery Plan for the Ozark Hellbender (*Cryptobranchus alleganiensis bishopi*). Bloomington, MN, USA: US Fish and Wildlife Service, Midwest Region.

[zoo21869-bib-0068] Vonesh, J. R. , and O. De la Cruz . 2002. “Complex Life Cycles and Density Dependence: Assessing the Contribution of Egg Mortality to Amphibian Declines.” Oecologia 133, no. 3: 325–333. 28466219 10.1007/s00442-002-1039-9

[zoo21869-bib-0069] Werner, E. E. 1986. “Amphibian Metamorphosis: Growth Rate, Predation Risk, and the Optimal Size at Transformation.” American Naturalist 128, no. 3: 319–341.

[zoo21869-bib-0070] Wheeler, B. A. , E. Prosen , A. Mathis , and R. F. Wilkinson . 2003. “Population Declines of a Long‐Lived Salamander: A 20 +‐Year Study of Hellbenders, *Cryptobranchus alleganiensis* .” Biological Conservation 109, no. 1: 151–156. 10.1016/S0006-3207(02)00136-2.

[zoo21869-bib-0071] Wilbur, H. M. 1976. “Density‐Dependent Aspects of Metamorphosis in *Ambystoma* and *Rana sylvatica* .” Ecology 57, no. 6: 1289–1296.

[zoo21869-bib-0072] Wilbur, H. M. 1977. “Density‐Dependent Aspects of Growth and Metamorphosis in *Bufo americanus* .” Ecology 58, no. 1: 196–200.

